# Acquired hemoglobin H disease in a patient with aplastic anemia evolving into acute myeloid leukemia

**DOI:** 10.1590/S1516-31802004000600009

**Published:** 2004-11-04

**Authors:** Maria Stella Figueiredo, Perla Vicari, Eliza Yuriko Sugano Kimura, Sandra Vallin Antunes, Mihoko Yamamoto

**Keywords:** Hemoglobin H, Disease, Aplastic anemia, Myeloid leukemia, Myelodysplastic syndromes, Doença, Hemoglobina H, Anemia aplástica, Leucemia mielóide, Síndromes mielodisplásicas

## Abstract

**CONTEXT:**

The prognosis of severe aplastic anemia has improved since the introduction of bone marrow transplantation and treatment with antithymocyte globulin. In contrast to the success of these protocols, studies with long term follow-up have shown the occurrence of clonal diseases such as paroxysmal nocturnal hemoglobinuria, myelodysplastic syndrome and acute leukemia in aplastic anemia.

**CASE REPORT:**

We report the first case of a Brazilian patient with aplastic anemia who developed myelodysplastic syndrome and acute myeloid leukemia showing acquired hemoglobin H and increased fetal hemoglobin.

## RESUMO

**CONTEXTO:**

O prognóstico da anemia aplástica grave melhorou com o advento do transplante de medula óssea e do tratamento imunossupressor com globulina antitimocitária. Em contraste com o sucesso destes protocolos, os estudos com seguimento a longo prazo mostraram a ocorrência de doenças clonais, tais como: hemoglobinúria paroxística noturna, síndrome mielodisplásica e leucemia aguda.

**RELATO DE CASO:**

Nós relatamos o primeiro caso descrito no Brasil de um paciente com anemia aplástica que evoluiu para síndrome mielodisplásica e leucemia mielóide aguda associada a presença de hemoglobina H e aumento da hemoglobina fetal.

## INTRODUCTION

The use of antithymocyte globulin as a single therapy or in combination with cyclosporin and corticosteroids has changed the natural history of severe aplastic anemia in patients who do not have a bone marrow donor. Today, antithymocyte globulin is a useful therapeutic tool with a response rate of 40-80%. However, despite the improvements achieved by using androgens, corticosteroids, cyclosporin and/or antithymocyte globulin, some quantitative abnormalities remain in all hematopoietic cell lines, thus suggesting that aplastic anemia has not been completely eliminated.^1^

Relationships have been described between aplastic anemia and acute leukemia, and with other clonal disorders such as paroxysmal nocturnal hemoglobinuria and myelodysplastic syndrome. The evolution of aplastic anemia to myelodysplastic syndrome or acute leukemia was considered to be rare (1-3%) in studies dating from the late 1950s to the 1970s, although a 57% risk of developing hematological complications at a time of eight years after antithymocyte globulin therapy was reported in 1988.^2^ We report a case of aplastic anemia with acquired hemoglobin H disease evolving into myelodysplastic syndrome and leukemia.

## CASE REPORT

A 36-year-old man presented with a 4-month history of pallor and fatigue. He had been working as mechanic for 10 years with frequent benzene exposure. Upon admission, he was pale, afebrile and his pulse rate was 100 beats/min. The peripheral blood cell count showed hemoglobin concentration of 2.8 g/dl, hematocrit of 9%, mean corpuscular volume of 82 fl, mean corpuscular hemoglobin mass of 27 pg, mean corpuscular hemoglobin concentration of 33 g/dl, white blood cell count of 1.8 X 10^9^/l, and platelet count of 5 x 10^6^/l. The reticulocyte count was 1% and Ham's test was negative. The bone marrow aspirate showed significant hypocellular marrow without dyserythropoietic features and the bone marrow biopsy was inconclusive. He was treated with prednisone and androgens (oxymetholone), which led to gradual improvement in the peripheral blood cell count, with low platelet count (45 x 10^6^/l).

After two years of follow-up, the patient maintained the same hematological pattern without further treatment. A second biopsy showed hypocellular marrow with erythroid hyperplasia.

Three years later, he complained of symptoms due to anemia. His hemoglobin concentration was 9.6 g/dl, with hematocrit of 30%, mean corpuscular volume of 80 fl, mean corpuscular hemoglobin concentration of 28 g/dl and reticulocyte count of 10.2%. Erythrocyte inclusions of "golf-ball" pattern, typical of hemoglobin H, were seen. Hemoglobin electrophoresis demonstrated the presence of hemoglobin H, with raised fetal hemoglobin of 8.4%. Bone marrow aspiration was again performed, showing erythroid hyperplasia, dyserythropoiesis with normal iron stores and absence of ring sideroblasts. The sucrose hemolysis test and Ham's test were negative.

**Figure 1:**
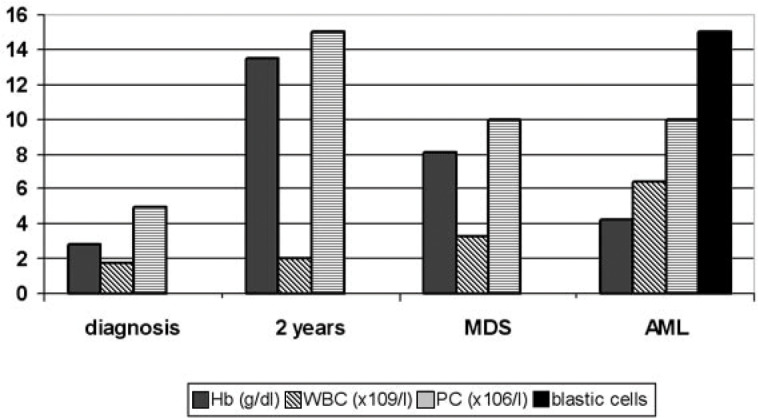
Haematologic changes during the course of the disease. Hb = haemoglobin; WBC = white blood cells; PC = platelet count; MDS = myelodysplatic syndrome; AML = acute myeloid leukaemia.

Ten months later, he was admitted with severe anemia (hemoglobin of 4.3 g/dl). The bone marrow aspirate demonstrated a blast cell concentration of 64%, and a diagnosis of acute myeloid leukemia was made (Figure 1). This was confirmed by immunophenotyping, but no cytogenetic study was performed. Induction therapy with daunorubicin and cytosine arabinoside was initiated, but remission was not achieved. He died after a second course of chemotherapy due to intracranial hemorrhage.

## DISCUSSION

Improvements in the treatment of aplastic anemia by means of bone marrow transplantation, and the use of antithymocyte globulin and/or cyclosporin, have raised the survival rate for this disease from 60% to 90% but, at the same time, the risk of late clonal hematological complications has increased.^1-3^

Few cases of aplastic anemia cases developing with acute myeloid leukemia or myelodysplastic syndrome have been reported in the literature.^1-3^ On the other hand it has long been commonly known that myelotoxic agents, such as radiation and benzene, can cause aplastic anemia as well as acute leukemia.

Hemoglobin synthesis disorders are frequently observed in the presence of clonal erythropoiesis. It has been suggested that disordered gene regulation is a common feature of transformed cells, and that this can be manifested either by the synthesis of an inappropriate gene product, such as fetal hemoglobin synthesis, or by the loss of an appropriate product, such as deficient production of an α-globin chain resulting in acquired hemoglobin H.^4^

Persistence or reactivation of fetal hemoglobin synthesis has generally been associated with certain dyserythropoietic conditions, such as allogenic stem cell transplantation, aplastic anemia, paroxysmal nocturnal hemoglobinuria, myelodysplastic syndrome or acute myeloid leukemia. However, the mechanism for fetal hemoglobinuria synthesis is unknown and the clinical value of fetal hemoglobin levels under these conditions remains controversial.^4^

There is a clear association between acquired hemoglobin H disease and clonal hematopoiesis, but frequency of such an association is unknown, probably because reticulocyte preparations are not set up or adequately examined for the presence of hemoglobin H inclusions.^4^

Molecular studies of acquired hemoglobin H disease have shown the presence of structurally normal α-globin genes but with decreased expression. It is not completely clear whether the presence of hemoglobin H is a marker for a particular subgroup of myeloproliferative disorders or whether it is a secondary event that may develop in neoplastic cells.^5^

A clear distinction between aplastic anemia and hypoplastic myelodysplastic syndrome must always be made, because they have different risks of developing with acute myeloid leukemia: 9% for patients with aplastic anemia and 83% for those with myelodysplastic syndrome.^6^ In our case, the bone marrow biopsies were not helpful in answering this question. However, our patient presented rapid improvement in hematopoiesis, upon therapy. Moreover, he did not need transfusion for 5 years. All these aspects support the hypothesis of aplastic anemia. After this period, he presented dyshematopoietic abnormalities and, subsequently, acute myeloid leukemia.

Tichelli et al.^2^ observed that rapid recovery of the bone marrow after aplastic anemia is the most predictive risk factor for transformation into leukemia. Although these dysplastic alterations are most prominent between 3 and 6 months after treatment, they may disappear and the bone marrow morphology may look normal for varying periods of time before leukemia occurs.^2^

Nowadays, aplastic anemia is considered to be a monoclonal disease that sometimes is an early manifestation of myelodysplastic syndrome or acute myeloid leukemia.^1^ Some studies have demonstrated the presence of monoclonal hematopoiesis in 72% of patients with aplastic anemia.^2,3,6^ Nevertheless, it is not known whether clonal hematopoiesis could have a prognostic value in aplastic anemia.

Finally, the appearance of late hematological complications in patients with aplastic anemia raises the question of whether myelodysplastic syndrome, paroxysmal nocturnal hemoglobinuria and aplastic anemia are just different manifestations of the same disease. We suggest that aplastic anemia and acquired hemoglobin H disease could be considered to be two steps in the neoplastic transformation of the pluripotent stem cell.
